# Advancement in Partograph: WHO’s Labor Care Guide

**DOI:** 10.7759/cureus.30238

**Published:** 2022-10-12

**Authors:** Yash Ghulaxe, Surekha Tayade, Shreyash Huse, Jay Chavada

**Affiliations:** 1 Medical Student, Jawaharlal Nehru Medical College, Datta Meghe Institute of Medical Sciences, Wardha, IND; 2 Department of Obstetrics and Gynaecology, Jawaharlal Nehru Medical College, Datta Meghe Institute of Medical Sciences, Wardha, IND

**Keywords:** women-centered care, labour, world health organisation, labour care guide, partograph

## Abstract

Worldwide, the partograph, also known as a partogram, is used as a labor monitoring tool to detect difficulties early, allowing for referral, intervention, or closer observations to follow. Despite widespread support from health experts, there are worries that the partograph has not yet fully realized its potential for enhancing therapeutic results. As a result, the instrument has undergone several changes, and numerous studies have been conducted to examine the obstacles and enablers to its use. Nevertheless, the partograph was widely embraced and has been a component of evaluating labor progress. Earlier it was also used as a standard method for monitoring labor progress. Even though it is widely used, there have been reports of usage and accurate execution rates. The WHO Labor Care Guide (LCG) was created so that medical professionals could keep an eye on the health of pregnant women and their unborn children during labor by conducting routine evaluations to spot any abnormalities. The tool intends to enhance women-centered care and encourage collaborative decision-making between women and healthcare professionals. The LCG is designed to be a tool for ensuring high-quality research centered on health, reducing pointless measures, and offering comfort measures.

## Introduction and background

Worldwide, more than 14 crore females have childbirth per year, and the percentage of deliveries by trained medical workers is rising gradually [1]. Complications during labor and delivery cause maternal deaths (>1/3rd), stillbirths (1/2), and newborn deaths (1/4th) [2,3]. Most of these fatalities occur in low-resource environments and might be largely avoided with prompt interventions [4].

Over the past 20 years, widespread support for skilled birth attendance has been made in an effort to reduce unnecessary maternal and perinatal death and morbidity [5]. Worldwide, this has resulted in significant increases in facility-based deliveries and the coverage of births attended by trained medical staff (up from 62% in 2000 to 80% in 2017) [6]. Although the range has increased, this has not necessarily resulted in the anticipated decline in mortality and morbidity during delivery, indicating that subpar treatment grade is still a problem in hospitals [7-11]. Regularly overused interventions include early amniotomy, oxytocin for augmentation, and continuous fetal monitoring [12]. Rising Caesarean section (CS) rates and poorer birth experiences for women have been attributed to this over-medicalized and frequently disrespectful treatment [13-15]. Therefore, it is critical to regularly monitor labor and delivery to spot dangers or difficulties and stop bad birth results. The most popular labor monitoring device is the partograph, and trained healthcare professionals have been using it to provide care during labor for more than 40 years.

However, the partograph needed to be revised to facilitate care following new research and international priorities in light of the WHO recommendations (2018) on intrapartum supervision for pleasant birth [16]. To promote the health and wellbeing of women and their unborn children, the guidelines build up recent development of definitions of the length of the first and second stages of labor. In addition, they offer advice on time and usage of labor interference [17, 18]. The WHO guidelines included in this list are: care during labor and delivery includes considerate maternal care, effective communication, labor amity, and continuity of care. The first stage includes clinical pelvimetry upon admission, the definition of latent and active first stages, the length and advancement of the first stage, the labor ward admission policy, pubic shaving, access enema, and vaginal examination. The second stage of labor includes: what it is, how long it lasts, how to give birth (with or without epidural anesthesia), how to push, how to avoid perineal injuries, and how to do an episiotomy. Prophylactic uterotonics, delayed cord clamping, controlled cord traction, and uterine massage are done during the third stage of labor. Regular nasal or oral suction during resuscitation, skin-to-skin contact, nursing, vitamin K prophylaxis for hemorrhagic sickness, bathing, and other early postnatal care of the infant are all included in the newborn's care. Following an uneventful vaginal birth, the lady will get postpartum care that includes monitoring her uterine tonus, taking antibiotics, standard postpartum maternal assessments, and discharge.

As a result, in 2018, WHO began work on a "next-generation" partograph, the WHO Labor Care Guide (LCG), with the following objectives: frequently refreshing professionals to provide reassuring treatment during delivery and to refresh for regular inspection that should be done at work to spot any developing complications, in the mother and the fetus; offer benchmarks for aberrant labor observations that should prompt particular responses; reduce needless interventional use and over- and under-diagnosis of problematic labor episodes; assist with audits and raising the standard of labor care. The old WHO partogram failed to demonstrate any meaningful clinical effect; hence this is an urgent requirement [19]. It is crucial that the LCG can accommodate maternity care and professionals' needs and that it contains the proper criteria for labor monitoring. The LCG was designed to care for mothers and their newborns throughout labor and delivery. Although there was no risk status, it comprises evaluations and examinations, which are crucial for treating all child-bearing females.

However, the LCG was primarily intended to be utilized to care for pregnant people who appeared to be in good health and their unborn children (i.e., low-risk pregnancies). Women at a greater risk of having difficulties during delivery might need more specific monitoring and care [20]. Even though LCG was primarily developed to be used for the surveillance of child-bearing females who appeared to be in good health, high-risk females who were having labor problems could also benefit from it as an observing tool [21]. Regardless of the woman's parity or the condition of her membranes, documentation on the LCG of the mother's and the baby's health and the labor's progression should begin when she enters the first stage's active phase of labor (five centimeters or greater cervical dilatation). Women and their babies are expected to be observed and provided care and support during the latent stage of labor, even though LCG should not be started at that time. Pregnancy, delivery, postpartum, and infant care: a guide for essential practice provides comprehensive instructions on how to treat patients during the latent period of labor [22]. The LCG was developed after extensive research, information synthesis, consultation, field testing, and improvement [23,24].

## Review

**Figure 1:**
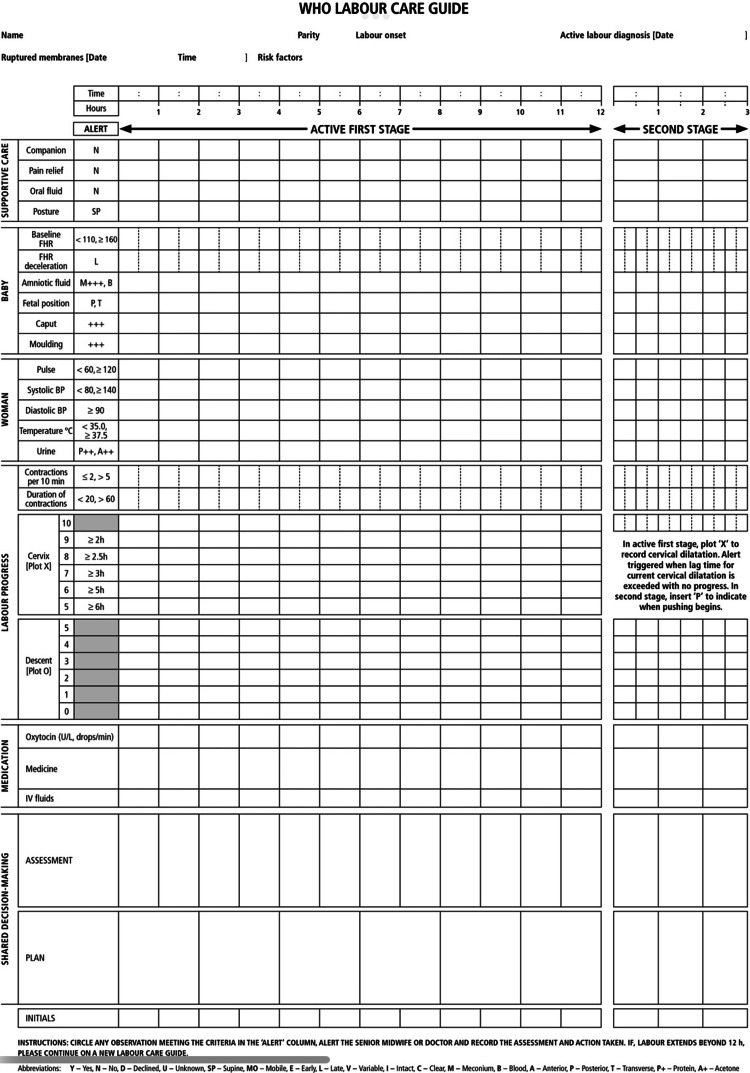
WHO's Labor Care Guide. Source: [25]

The LCG differs from earlier partograph designs in addressing the length of labor, identifying when clinical interventions are necessary, and focusing on keeping the mother safe. It is expected that a change from common partograph makeup may make medical professionals uneasy or even hostile. However, change should not be implemented merely for the sake of change because it is difficult. Therefore, the seven portions of LCG were modified from the original partograph layout. As shown in Figure 1, the sections are: identifying information and labor characteristics at admission, supportive care, care of the baby, care of the woman, labor progress, medication, and shared decision-making.

The woman's name, parity, method of labor onset, date of active labor diagnosis, date and time of membrane rupture, and risk factors should all be recorded in Section 1 of the labor admission record. This part should be finished with the knowledge acquired after a confirmed diagnosis of active labor. There is a list of labor observations in Sections 2-7. As soon as the lady is admitted to the labor unit, the healthcare provider should start recording observations for all parts. The remaining LCG is then finished after additional labor-related evaluations. Each compliance has two axes, a perpendicular line data axis for noticing any departure from the typical remarks and a horizontal time axis for tracking the length of the inquiry. The woman's name and other crucial details necessary to comprehend her baseline features and risk status at the time of labor admission are written in Section 1 as well. The woman's medical file should also have information on other crucial demographic and labor features, like her age, period of gestational, serological outcomes, hematocrit, blood group, Rh factor, status, referral reason, and symphysis-fundal length. Supportive care is discussed in Section 2. The WHO's recommendations for intrapartum care strongly emphasize respectful maternity care as a fundamental human right of expectant mothers [26]. At each stage of labor care, the WHO advises clear interaction between health professionals and women in labor, including using easy, appropriate terminology. All women should receive a thorough explanation of the techniques being used and why. The woman and her companion should be informed of the results of the physical examinations. Baby care is included under Section 3. The fetal heart rate (FHR), amniotic fluid, the position of the fetus, the shape of the fetal head, and the development of the caput succedaneum are all regularly observed to determine the baby's health. Section 4 adds maternal care. This section attempts to make it simpler to make decisions on sporadic, ongoing monitoring of women's wellness. On the LCG, the pulse, blood pressure, temperature, and urine are regularly observed to keep track of the woman's health and well-being. Work progress is shown in Section 5. This part promotes the routine practice of periodic observation of labor development markers. The labor process is tracked on LCG by regularly observing the frequency and length of contractions, cervical dilatation, and fetal descent. By indicating the patient is getting oxytocin, its dosage and whether other drugs or IV fluids are given. Section 6 intends to allow constant observation of all forms of drugs used during labor. Section 7 makes it easier for the lady and her companion to communicate continuously and for all assessments and agreed-upon plans to be consistently recorded.

The partograph and the LCG are tools used to enhance women-centered care, but they also have some points in common and distinctions. The fundamental and revolutionary aspect of the original "Philpott chart" was its graphical display of labor development in relation to women's cervical dilation and fall of presenting part of the fetus against time [27,28]. The LCG and modified WHO partograph share some of these characteristics. Despite cosmetic changes, this idea still holds a prominent place in the LCG. Additionally, regular formal monitoring of significant clinical parameters reflecting the frequency and duration of uterine activity and the health of the mother and the unborn child continues. The LCG mainly focuses on clinical parameters rather than parameters obtained from USG [25]. The LCG has the following improvements among the two uncommon features: the initial moment of the first stage's active phase of labor is dilatation of the cervix of five centimeters (in spite of four centimeters or less); it adds up a division for observing second labor stage; it consists of a division to evaluate and advance the use of understanding interruptions to enhance child health, and every cm of cervical dilatation during the first stage of labor resulted in a shift in the hourly "alarm" line and its corresponding "action" line with corroboration-based time constraints. Table 1 below provides information on the frequent and rare characteristics of LCG and modified partograph [29]. Specialists distinguished a few difficulties in utilizing LCG that are normal to the utilization of any partograph. A 2014 deliberate survey on partograph use in low-pay and center-pay nations found that while the partograph was largely seen as a valuable device for observing work, its utilization was frequently seen as tedious [30].

**Table 1:** Difference in features between modified partograph and LCG. LCG: Labor Care Guide.

Modified Partograph	Labor Care Guide
The active phase begins to start at cervix dilation from 4cm	The active phase begins to start at cervix dilation from 5 cm
Alert and action line fixed at 1 cm/hr	Proof-based time restrictions on cervical dilations at each centimetre
Keeps track of the intensity, frequency, and length of uterine contractions	Keeps track of span and repetition of uterine shrinking
There is no second stage	Enhanced surveillance in the second stage
No documentation of interventions for reassuring help	Companionship during labour, pain alleviation, oral fluid intake, and posture are all explicitly recorded
Besides cervical dilatation warning and action lines, there is no clear necessity to act in response to observation that differ from predictions of any labour	Requires that observation be noted, along with the provider recording the appropriate response

Common features are animated timelines showing the progression of labor as measured by cervical dilatation and the head descent and formal and consistent documentation of crucial clinical indicators indicating the mother's and child's health. However, there were some restrictions on how typical labor progressed. Since labor's active first stage is supposed to be indicated by a line drawn at one centimeter per hour from the initial assessment of the cervix, the original partograph uses this technique (three or four cm) to identify prolonged labor (when the action line is crossed), and a parallel line two hours (commonly four hours) later, as the alert or projected normal progress line [31]. This style was developed using Friedman and Kroll's landmark research, suggesting the average cervical dilatation rate in primigravida was diphasic, slower before three-centimeter dilatation, and roughly one centimeter per hour after three centimeters [31]. The primary problem with converting this statistical overview of several labors into a template for specific women is that it ignores the variation in women's progression rates. Additionally, because the action line criterion for protracted labor is set in advance for full delivery, it does not consider the non-linear progression of each woman's labor. For instance, it might take longer than four hours to get to the action line if labor had advanced quickly and was suddenly stopped. However, suppose labor has been prolonged due to insufficient uterine movement and has traversed the action line. In that case, it might continue to advance usually but cause anxiety as it is on the action line's wrong side. This anxiety is then useless for directing the course of the rest of the labor. Because guiding factors for labor advancement are dynamic rather than static in the new table for recording labor advancement in LCG, there are significant differences. Instead of setting constant rate boundaries for overall labor's active first stage, contemplation for interruption is governed by a proof-based limit of time for every inch of dilatation of the cervix, which is acquired from 95th percentiles of labor span at various cm stage in a female with ordinary perinatal results [32]. Even if it takes an unusually short amount of time to reach a dilation of 9 cm, the projected upper limit of 10 cm stays the same. The appropriate cervical assessment (designated by an x) would be highlighted. Actions for responding will be recorded in the plan division, just as in different metrics in the alert division, such as moving from nine to ten centimeters and exceeding two hours. LCG and the partograph vary most noticeably in not presence of the limit lines (diagonal labor progress). An obvious need for recorded feedback when certain parameters have crossed, even though lines are discarded, and the parameters are now presented in a modern, proof-based fashion. The definition of the active phase depends upon the point of inflection on the Friedman curve, original partograph during the first stage of labor identified the beginning of the active phase as 3 cm cervical dilation. The WHO shifted this threshold to 4 cm due to changes to the partograph [33]. It is compelling that Friedman lately pointed out a misinterpretation of his project and admitted that point of inflection does, in fact, vary from woman to woman. The three or four-centimeter threshold was frequently elaborated as explaining Friedman's actual project. The median cervical dilation rate in normal women without unfavorable perinatal results was observed to pass one centimeter/hour at five centimeters, which marks the fast start of the cervical dilatation process and is the milestone used by the LCG [34]. This reduces the premature classification of labor's active phase, a significant elicited reason for ostensibly slow labor advancement and needless interruptions [35]. Because it can only be accurately identified in hindsight, the latent phase of the initial labor stage is challenging to identify, according to the LCG. Its timing is frequently ambiguous, and the length of time it lasts varies greatly across women. Innate in the real plan of the partograph, which set aside the latent phase for eight hours, is a potential source of unnecessary intervention known as premature charting of the latent phase. By starting to chronicle the progress of the labor process after the active phase has been identified, this is neglected in the updated partograph style and LCG. 

At a later stage of labor, the fact that the second stage of work is excluded from the original partograph design and its revisions is a significant drawback. During the labor's second stage, there is no obligation to explicitly continue keeping an eye on the mother's and the baby's health or the labor's progress. The second stage of work is particularly crucial because of increased uterine activity and the mother's efforts to expel the baby; if caution is not exercised during this time, disastrous results may result. The LCG addresses this gap by emphasizing the importance of paying near awareness to the development and the welfare of both mother and child during the second stage. The LCG is intended for the significance of the exploratory aspect of labor by demanding graphic noting of proof-based applications that are important for better clinical results for mothers and their children and women's good birth experiences. The LCG contains assessments of the labor partner, mouth hydration, mother motility and attitude, and pain management to promote intrapartum care and encourage using these evidence-based, but sometimes underutilized interventions. The LCG is beyond just a scientific instrument for keeping track of a woman's health and that of her unborn child throughout labor. The tool also offers testimonial values for the mother and fetal scrutiny and stimulates detailed documentation of the mother's vital signs, fetal welfare, and labor advancement. The plain necessity to circle any statement that conflicts with care, comfort, or labor advancement and to document clinical or reassuring care feedback in conferences with women serves to reinforce the tool's care purpose by encouraging early recognition and improvement of the support that mother and offspring experienced. The caregiver enters the overall evaluation, any new information not before recorded but crucial for labor observation is entered in the "Assessment" part, and the care scheme developed in consultation with the mother is entered in the "Plan" section. This makes LCG more than just labor documentation that could have been finished in the past; it makes it a contemporary monitoring and response tool. 

## Conclusions

In the past few years, a lot has shifted in the way we provide proof-based, compassionate care during delivery. Future studies will be needed to count women's experiences with care to completely comprehend the applicability and implications of labor care and results. Nevertheless, medical practitioners will be convinced that the fundamental principles that informed the construction of the modified partograph in utilizing the new instrument would not compromise but rather advance the objectives of the earlier partograph. LCG has evolved to reflect these changes and will motivate best practices, which add advancement of excellent, considerate care for all women, new mothers, and their families.
